# Genetic and molecular underpinnings of atrial fibrillation

**DOI:** 10.1038/s44325-024-00035-5

**Published:** 2024-12-04

**Authors:** Mason E. Sweat, WIlliam T. Pu

**Affiliations:** 1 Department of Cardiology, Boston Children’s Hospital, Boston, MA USA; 2 Harvard Stem Cell Institute, Harvard University, Cambridge, MA USA

**Keywords:** Cardiovascular genetics, Atrial fibrillation

## Abstract

Atrial fibrillation (AF) increases stroke and heart failure risks. This review examines genetic and molecular mechanisms underlying AF. We review genes linked to AF and mechanisms by which they alter AF risk. We highlight gene expression differences between atrial and ventricular cardiomyocytes, regulatory mechanisms responsible for these differences, and their contribution to AF. Understanding AF mechanisms through the lens of atrial gene regulation is crucial for developing targeted AF therapies.

## Introduction

In atrial fibrillation (AF), the most common sustained arrhythmia^1^, chaotic atrial electrical activity sustained by re-entrant circuits, replaces normal rhythmic atrial depolarization and contraction initiated by the sinus node. The chaotic electrical activity causes uncoordinated atrial contractions and the ineffective movement of blood through the atria. In addition, fibrillating atria often exhibit morphological remodeling, including dilatation and fibrosis^2,3^.

Patients with AF have elevated risk for stroke (5-fold)^4,5^ heart failure (HF; 1.7-fold) and death (3.7-fold)^6–8^. The worldwide number of individuals with AF was estimated to be 46.3 million in 2016^9^, and the prevalence will increase with increasing mean population age^10^. The lifetime risk for the US population is estimated to be 1 in 3 for individuals of European ancestry^11^. The disease represents an ever-increasing burden on the healthcare system, adding nearly $25,000 to the annual cost of care per person^12^. Despite its high socioeconomic cost, therapies to treat AF remain insufficient. Currently, available treatment options attempt to control heart rhythm or rate but have variable efficacy and significant risk for adverse effects.

We are entering into a new era for the treatment of myopathies and arrhythmogenic conditions, with novel treatments being developed to target underlying molecular and genetic mechanisms. For example, a treatment approach for hypertrophic cardiomyopathy through inhibiting (β)-myosin heavy chain ATPase activity recently gained FDA approval^13^, and gene therapies are currently being developed to treat the genetic lesions underlying HCM^14,15^. Similarly, elucidating the molecular basis for other muscle diseases and proarrhythmic conditions including Duchenne muscular dystrophy, Barth syndrome, catecholaminergic polymorphic ventricular tachycardia (CPVT), and arrhythmogenic cardiomyopathy has resulted in the development of gene therapies that have shown efficacy in preclinical or clinical trials^16–20^. To make AF similarly targetable, we must continue to advance our understanding of AF pathophysiology at the molecular and genetic levels.

In AF, one barrier to developing targeted therapies is an incomplete understanding of the aCM-specific gene regulatory network, the regulatory circuitry that aCMs use to express genes at the appropriate levels to maintain normal atrial contraction and rhythm. This network includes transcription factors, chromatin epigenetic modifications, and three-dimensional chromatin architecture. aCMs are phenotypically distinct from ventricular cardiomyocytes (vCMs)^21–23^, and these phenotypic differences are driven by the differential expression of thousands of genes^21,24^. Since aCM characteristics have been tuned to maintain atrial rhythm, comparing aCMs with vCMs can identify aCM properties, genes, and regulatory modules critical for maintenance of atrial rhythm.

This review will examine differences between aCMs and vCMs, including their unique developmental trajectories, ultrastructures, and gene expression profiles in mouse and human tissues, in order to identify features of aCMs that might be important for atrial rhythm homeostasis. Genes linked to AF by genome-wide association studies (GWAS), studies of AF families, and targeted sequencing of AF patients are then examined in the context of the aCM and vCM gene regulation and AF pathophysiology.

## aCM- and vCM-distinguishing features

Although aCMs and vCMs are both cardiomyocytes, each cell type has specific distinguishing features. Because aCM- and vCM-selective features are likely critical for proper atrial and ventricular function, understanding them and the regulatory mechanisms that maintain them could provide important insights into their dysfunction in disease and potentially lead to future therapies. We consider differences in physiological characteristics and developmental programs and examine genes differentially expressed between aCMs and vCMs in mice and humans.

### Chamber-specific differences in cardiomyocyte organization and function

**Fig. 1:**
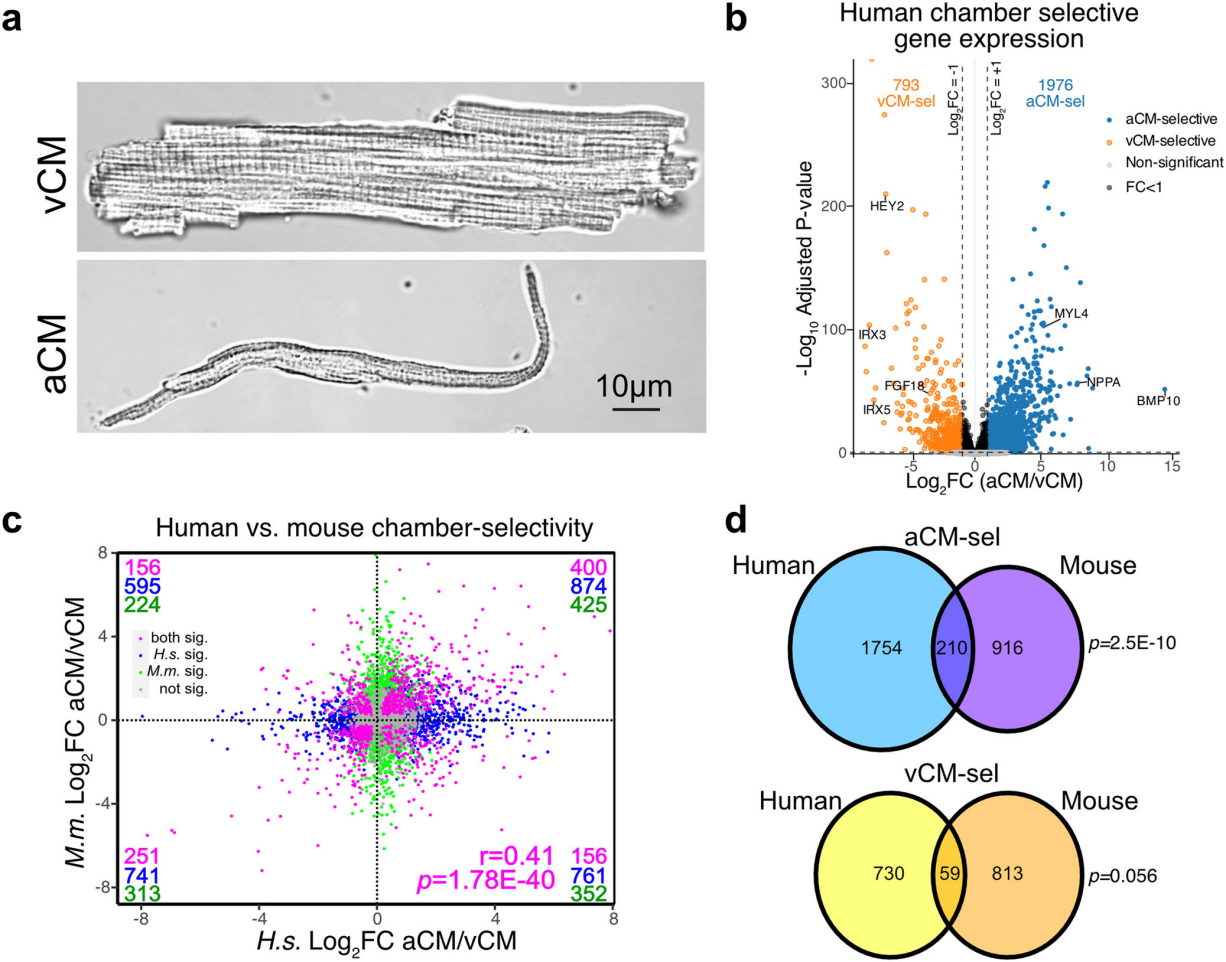
aCM and vCM-specific features. **a** Micrographs of isolated adult mouse ventricular and atrial cardiomyocytes. **b** Volcano plot obtained by pseudobulk RNA-seq analysis obtained from the human heart atlas. aCMs and vCMs were extracted from samples with >500 aCM or vCMs, pseudobulked, and compared using DEseq2. Genes with chamber selectivity were considered with Log2FC > |1| and pval adj. < 0.05. **c** Human and mouse data were plotted against each other to examine species-specific biases in chamber selectivity. The Pearson correlation coefficient (*r* = 0.4, *p* = 1.78E − 40) was determined for genes that were differentially expressed in both datasets. **d** Intersection between genes selectively expressed in human or mouse atria or ventricles.

aCMs and vCMs differ in size, ultrastructural organization, metabolism, and electrical function^21–23^. The most obvious difference is their physical size; compared to vCMs, aCMs are both shorter and thinner (Fig. 1a)^21^. Perhaps related to their smaller size, aCMs have a lower density of T-tubules, invaginations of the plasma membrane that coordinate excitation–contraction coupling, than vCMs. Another contrasting feature of aCMs and vCMs is their metabolism. vCMs contain a much greater density of mitochondria^21^. In keeping with this observation, vCM-selective genes are enriched for functional terms related to oxidative phosphorylation and mitochondrial function^21^.

Electrically, aCMs exhibit less polarized resting membrane potentials and faster action potential kinetics than vCMs. More rapid membrane repolarization occurs in aCMs due to inward-flowing potassium currents found selectively in aCMs, including the atrial-specific ultra-rapid repolarization current (*I*_Kur_) mediated by the KCNA5 channel^25^ and the acetylcholine-activated K^+^ current (*I*_KACh_), mediated by the KCNJ3/5 channels^26^. These ion channels are expressed at greater levels in aCMs compared to vCMs, resulting in the characteristic action potential shape of each cardiomyocyte subtype.

### aCM and vCM-selective gene expression

Gene expression profiles differ between aCMs and vCMs. These chamber-selective expression profiles include transcription factors important for the maintenance of aCM or vCM identity (discussed in greater detail below), and altering the expression of these key transcription factors can blur the distinct phenotypes of aCMs and vCMs. For example, inactivating *Nr2f2* in early development resulted in a denser and more organized T tubule network in cardiomyocytes of the atrial chamber^27^, a vCM-like feature, while *Tbx5* inactivation in aCMs increased action potential duration^28^, making them more similar to vCMs.

Several sarcomeric genes are differentially expressed between aCMs and vCMs, including the aCM-specific myosin light chains 4 and 7 (*Myl4* and *Myl7)* and the vCM-specific *Myl2* and *Myl3*. There are other well-known examples, including *Nppa*, which is initially expressed in all cardiomyocytes, becomes restricted to postnatal aCMs, and is re-expressed in postnatal vCMs under pathological stress^29^. While these markers represent some of the most highly differentially expressed aCM and vCM-selective genes, many other genes are differentially expressed to a lesser degree. Early comparison of gene expression differences between atria and ventricles employed whole tissues^30,31^, which obfuscated the comparison between cardiomyocyte subtypes. Recently, we used bulk RNA sequencing to measure gene expression differences between isolated neonatal murine aCMs and vCMs^21^. We detected 1126 genes with aCM-selective gene expression and 872 genes with vCM-selective gene expression (|Log_2_FC| > 1.5, Padj < 0.05).

Although mice and humans share many aspects of cardiac biology, there are also notable differences, including differences in heart rate and a subset of sarcomere and ion channel genes^14^. To compare differences in gene expression between human aCMs and vCMs, we analyzed publicly available snRNAseq datasets of non-diseased adult human atria and ventricles^32,33^. Pseudobulk RNA sequencing analysis of cardiomyocyte nuclei from these tissues^33^ identified human aCM- and vCM-selective genes (|Log_2_FC| > 1 and Padj < 0.05) (Fig. 1b). Like mice, human aCMs had more chamber-selective genes (1976) than vCMs (793, Supplementary Table 1). We compared aCM vs. vCM expression differences between species (Fig. 1c). Despite the numerous technical and experimental differences between these analyses, twice as many genes were concordant between species (quadrants I and III) than discordant (quadrants II and IV), and genes with chamber-selective expression in both species were moderately but highly significantly correlated (Pearson *r* = 0.4, *p* = 1.78E-40). This analysis demonstrates the cross-species conservation of many chamber-specific characteristics. However, many genes exhibited selective expression in only one species. Additional experiments will be required to determine if these divergent genes are biological or attributable to technical differences between these studies. These data support the use of mice to study chamber-specific gene expression and diseases but suggest species-specific differences might complicate the cross-species interpretation of some results.

To circumvent the limitations of mice to model human disease, protocols have been developed to selectively differentiate human pluripotent stem cells (PSCs) into aCMs or vCMs^34,35^. These protocols, which largely depend on the addition of retinoic acid during differentiation to promote aCM as opposed to vCM specification, were recently reviewed^36^. While largely successful in producing PSC-aCMs with atrial-like action potential kinetics and gene expression profiles, some limitations include high spontaneous beating frequency, an immature phenotype, and the expression of *SHOX2*, a marker of the sinoatrial node lineage. More recent studies combined the co-culture of PSC-aCMs with primary human adult atrial cardiac fibroblasts and soft lithographic patterning of ECM proteins in order to obtain more mature aCMs^37^. However, this process is laborious and technically demanding, creating the need for more streamlined approaches.

In spite of their limitations, PSC-aCMs have been successfully applied to study several AF-causing variants^38–40^. Ghazizadeh et. al. studied PSC-aCMs harboring a variant in the atrial-selective gene *MYL4* that segregated with familial AF in an Icelandic family^41^. This approach revealed multiple cellular mechanisms promoting AF, including increased retinoic acid production and actin disorganization. Increased protein kinase C activity, downstream of increased retinoic acid signaling, elevated phosphorylation, and channel permeability of the gap junction protein CX43. A drug screen in the PSC-aCMs and a zebrafish model harboring the same variant showed that the small molecule carbenoxolone, a CX43 inhibitor, reversed these phenotypes. This work highlights the use of in vitro modeling approaches to dissect AF mechanisms and identify potential therapeutic approaches. It also illustrates the value of approaches to produce and characterize PSC-aCMs.

### Development and maintenance of aCMs and vCMs

During heart development, aCMs and right ventricular vCMs arise from second heart field progenitors, whereas left ventricular vCMs arise primarily from first heart field progenitors. aCMs and vCMs proceed through specialized developmental programs resulting in the two distinct but related cell types^27,42–48^. Following heart looping, the primitive atria and ventricles are demarcated by the selective expression of transcription factors, signaling pathway proteins, and cardiomyocyte functional components specific to aCMs and vCMs. One of the earliest identified chamber-selective transcription factors, IRX4, is restricted to the developing ventricles, and its ectopic overexpression in embryonic chick atria induced the expression of a vCM-specific myosin^44^. Since this initial discovery, multiple transcription factors and signaling pathways contributing to the vCM developmental program have been identified, including FGF signaling and the transcription factors NKX2-5/2-7 and HEY2^43,46,47^.

Several factors have been shown to promote aCM differentiation. Retinoic acid (RA) signaling is critical for determining the posterior second heart field (pSHF), which gives rise to most of the atrial compartment^36^. In addition to retinoic acid signaling, the transcription factor *Nr2f2* promotes atrial differentiation^27^. *Nr2f2* inactivation at E12.5 resulted in the downregulation of *Myl7*, an atrial-specific myosin light chain, and upregulation of *Myl2*, its ventricle-specific counterpart. However, the inactivation of *Nr2f2* later in development failed to alter aCM identity, suggesting distinct requirements for aCM differentiation vs. maintenance, with *Nr2f2* required for the former and not the latter.

Cell identity is maintained through the regulation of gene expression by tissue-specific enhancer–promoter networks. Recent studies by our group examined transcription factors important for the postnatal maintenance of either aCM or vCM-selective gene expression^21,24^. We used a massively parallel reporter assay to identify enhancer elements with selective activity in aCMs and vCMs. Motif analysis suggested that the estrogen-related receptor (ERR) and TBX5 were important vCM-selective and aCM-selective enhancer activity, respectively. Inactivating ERRα and ERRγ in postnatal mouse hearts resulted in the downregulation of many vCM-selective genes and upregulation of many aCM-selective genes, confirming that ERRα/γ are essential to maintain vCM gene expression.

TBX5 is a transcription factor that is initially expressed in the cardiac crescent^49^. Expression soon becomes restricted to the posterior portion of the heart tube, which will develop into the atria. As the heart loops and septates, this graded expression is maintained, with highest expression in atria, moderate expression in the left ventricle, and little to no expression in the right ventricle and outflow tract. TBX5 is also expressed in the AV node and cardiac conduction system, where it is essential for cardiac conduction system specification and gene expression^50^. In the mature heart, *Tbx5* expression is ~10-fold higher in aCMs than left ventricular vCMs, and postnatal inactivation of *Tbx5* in aCMs caused AF^23,24,28,51^, which was accompanied by downregulation of aCM-selective genes and upregulation of vCM-selective genes^24^. Upon *Tbx5* inactivation, regions near aCM-selective genes that are normally bound by TBX5 became less accessible, had diminished H3K27ac, an active enhancer mark, and lost contacts with promoters^24^. Increased TBX5 activity also causes AF, as a *TBX5* missense gain of function mutation caused familial AF, and this was recapitulated in mouse models^52,53^. Together, these data demonstrate that TBX5 is required to maintain aCM identity and atrial rhythm.

Notably, altering the chamber-selective expression programs of either aCMs or vCMs is detrimental to heart function. Mice lacking cardiomyocyte ERRα and ERRγ develop bradycardia and lethal cardiomyopathy^54^, while mice lacking TBX5 in aCMs develop permanent AF^24,28^, atrial cardiomyopathy, and at later stages, fibrotic remodeling restricted to the atrial chambers (ref. ^24^ and our unpublished observations). These observations suggest that maintaining chamber-selective cardiomyocyte identity is critical for healthy heart function.

## AF genetics

AF demonstrates high heritability, pointing to a strong genetic component. A previous study of Danish monozygotic and dizygotic twins suggested that AF heritability was as high as 62%^55^. However, family-based studies may overestimate heritability, and a more recent study estimated that AF heritability is 22.1%, with the large majority attributable to the additive effects of common genetic variants (minor allele frequency > 5%)^56^. In addition to common variants with smaller effect sizes, rare variants with larger effect sizes are also observed. Lone AF (AF in the absence of structural heart disease) aggregates within families^57^. In the Framingham Heart Study, 30% of offspring participants that developed new-onset AF had at least one parent with AF, and an AF diagnosis in at least one parent was positively associated with developing AF (OR, 1.85, *p* = 0.02)^58^. Another study in the Icelandic population demonstrated that an AF diagnosis in individuals under 60 years of age was more than five times as likely if they were a first-degree relative of a family member diagnosed with AF prior to the age of 60^59^. Together, these studies highlight the substantial genetic components underlying AF susceptibility.

### Identifying genes associated with AF pathogenesis through genome-wide association studies

GWAS, linkage analysis, and coding variation have been used to determine the genetic underpinnings of AF. The first AF GWAS, reported in 2007, examined a cohort of 550 patients vs. 4476 controls. This study identified two single nucleotide polymorphisms (SNPs) in the 4q25 locus near the transcription factor *PITX2*^60^. Since then, GWAS studies have expanded dramatically in study population size and hence statistical power^61–69^, with the most recent study^69^ comparing 77,690 cases from Japan and Europe against 1,167,040 controls. From this analysis, 150 loci with genome-wide significance (log_10_ Bayes factor (BF) > 6) were identified, including 33 novel loci. Among the 3637 genetic variants in linkage disequilibrium (*r*^2^ > 0.8) with the lead SNP of each locus, only 19 are missense variants in protein-coding genes, demonstrating that the majority of AF variants are found in the non-coding genome. Presumably, these non-coding variants alter the transcriptional regulation of their target genes.

To translate the discovery of AF risk variants into new therapies, it is essential to decipher the gene(s) that they regulate. This process is complicated by the fact that cis-regulatory regions do not necessarily regulate the closest gene or even a directly neighboring gene. Historically, genes have been associated with SNPs through expression quantitative trait loci (eQTL) analysis^70^. This method has now been augmented with molecular biological approaches that more directly implicate non-coding variants with target gene expression, including mapping enhancer–promoter contacts and epigenetically modulating candidate loci using CRISPR-i/a^71–73^ and CRISPR genome editing^74^.

Recent studies utilized these approaches to identify genetic drivers of AF^75–78^. van Ouwerker et al.^75^ identified candidate genes located within 1.9 mb of 104 genomic loci implicated in AF by (1) their presence within the same topologically associated domain; (2) contact of the implicated region with the gene promoter, as determined by promoter-capture Hi-C; (3) expression level in adult and fetal left and right whole atrial tissue and aCMs from adult left atria; and (4) eQTL analysis. If a gene was expressed, it received a score of at least 10, and a score of 11 or greater was considered a causative gene. This approach identified 264 potentially causative genes. However, a weakness of this study is that the scoring system lacked a strong statistical foundation^78^.

Another recent study^77^ used single nucleus RNA and ATAC sequencing (snRNAseq and snATACseq) to identify regulatory elements specific for each cardiac cell type. Region/gene associations were determined through co-accessibility analysis of the putative enhancers and target gene promoters^79^. Next, candidate cis-regulatory elements (CREs) specific for aCMs and vCMs were intersected with AF SNPs determined to be causative by Bayesian fine mapping (posterior probability of association >10%), resulting in the identification of 40 fine-mapped variants within 38 cardiac CREs and linked to 38 putative genes. The authors went on to validate one variant predicted to regulate *KCNH2*. The variant allele decreased enhancer activity in a luciferase assay, and CRISPR-mediated genomic deletion of the enhancer containing the variant reduced *KCNH2* mRNA levels and altered electrophysiological properties. This approach was successful but only prioritized genes at a small number of AF-associated loci.

Selewa et al. identified genes regulated by AF SNPs by using cardiomyocyte chromatin features (open chromatin, H3K27ac, and promoter-capture Hi-C contacts) to nominate associated cardiomyocyte enhancers^78^. These were linked to genes using a pipeline called ‘Mapgen’ to determine a Posterior Inclusion Probability (PIP) score for each gene surrounding each fine-mapped SNP based on the likelihood that the SNP regulated the gene. This likelihood was assigned a maximal value if it met any of the following criteria: (1) the SNP contacted the gene promoter, based on iPSC-CM promoter-capture Hi-C; (2) the SNP was located within an open chromatin region and within 20 kb of the gene; or (3) the SNP was located within the gene’s untranslated regions. If a SNP did not meet any of these criteria, the likelihood was determined by the distance from the SNP to the gene transcriptional start site, which decayed exponentially. Next, a ‘gene PIP score’ was determined by considering individual PIP scores for each of the SNPs predicted to target a given gene. Thus, if multiple SNPs targeted a single gene with high PIP scores, the gene was considered likely to drive AF and received a high gene PIP score. This approach resulted in the identification of SNP-gene pairs that captured >80% of the causal signal for each of the 122 loci examined, pinpointing a single gene at 42 loci and two genes at 34 loci. A total of 48 genes were considered highly likely (PIP > 0.8) by this approach. For several genes, high gene PIP scores were driven by aggregating multiple SNPs with moderate PIP scores. To validate their model, the authors demonstrated that several SNPs altered enhancer activity in luciferase assays in aCM-like HL-1 cells. While this study describes a powerful new approach for identifying potentially causal genes, it was based on vCM chromatin feature maps even though these features differ between aCMs and vCMs, especially near genes that are selectively expressed between the two cell types^77^. Scoring likely would be improved by using aCM-specific chromatin feature maps.

Experimental validation of informatic predictions remains a major bottleneck, both for validating the impact of SNPs on candidate gene expression, and for validating the significance of gene expression changes on AF pathogenesis.

### Features of genes implicated in AF

**Fig. 2:**
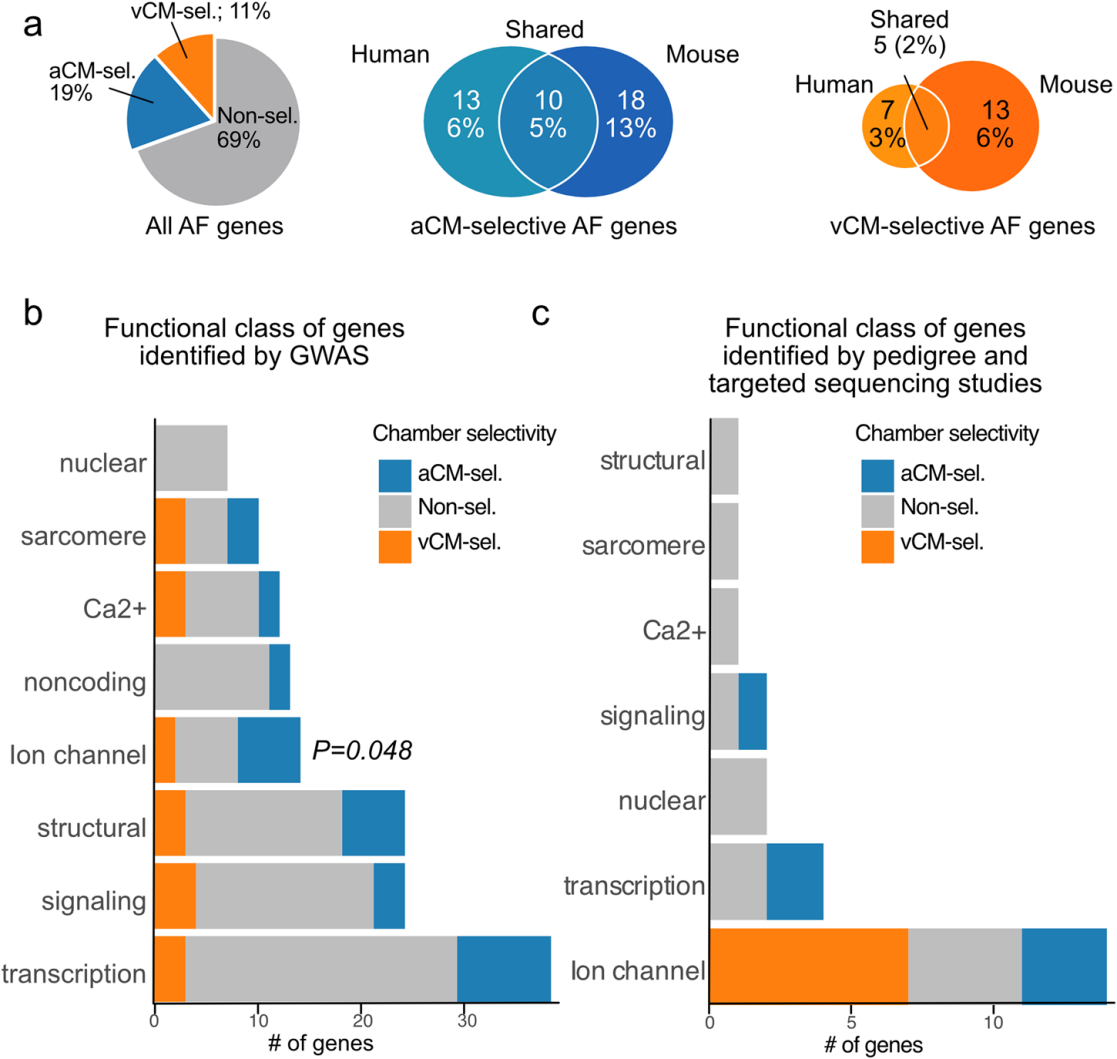
Characteristics of genes linked to atrial fibrillation. **a** Chamber-selective expression of atrial fibrillation genes. **b** Functional class of atrial fibrillation genes identified by GWAS. **c** Functional class of atrial fibrillation genes identified in pedigree and targeted sequencing studies.

We summarized the chamber-selective expression of the genes linked to AF by their proximity to AF GWAS loci or by Mapgen (Table 1). AF-linked genes had a higher propensity to be aCM-selective (Fig. 2a). In total, 41 (19%) AF genes were aCM-selective: 13 (6%) in humans, 18 (13%) in mice, and 10 (5%) in both. On the other hand, 25 (12%) genes showed vCM selectivity in one or both species, with 7 (3%) vCM-selective in humans, 13 (6%) in mice, and 5 (3%) in both. Although these genes were more highly expressed in vCMs, they were also expressed in aCMs, where their expression level is likely important for normal atrial rhythm.

**Table 1:** SNPs and genes implicated in AF by GWAS

rsID	Position (hg19)	Prioritized gene	Original citation	Category	Chamber selective (M.m)	Chamber selective (H.s.)
rs55985730	chr7:128417044	CALU	Nielsen et al.^68^	Ca^2+^		
rs4073778	chr1:116297758	CASQ2	Roselli et al.^67^	Ca^2+^		
rs3951016	chr6:118559658	PLN	Christophersen et al.^65^	Ca^2+^	vCM	
rs6829664	chr4:114448656	CAMK2D	Roselli et al.^67^	Ca^2+^		
rs6747542	chr2:70106832	ANXA4	Christophersen et al.^65^	Ca^2+^		vCM
rs7126870	chr11:3890059	STIM1	Miyazawa et al.^69^	Ca^2+^		vCM
rs11614295	chr12:113196733	RPH3A	Miyazawa et al.^69^	Ca^2+^		
rs2288327	chr2:179411665	FKBP7	Nielsen et al.^68^	Ca^2+^	aCM	
rs11841562	chr13:22111521	MICU2	Miyazawa et al.^69^	Ca^2+^		
rs7225165	chr17:1309850	YWHAE	Nielsen et al.^68^	Ca^2+^		
rs1278493	chr3:135814009	PPP2R3A	Nielsen et al.^68^	Ca^2+^		
rs12188351	chr5:168386089	SLIT3	Nielsen et al.^68^	Ca^2+^	aCM	aCM
rs11264280	chr1:154862952	KCNN3	Ellinor et al.^61^	Ion channel	vCM	aCM
rs35349325	chr12:70097464	BEST3	Roselli et al.^67^	Ion channel	aCM	
rs6790396	chr3:38771925	SCN5A*	Roselli et al.^67^	Ion channel		
rs4977397	chr9:20235004	SLC24A2	Roselli et al.^67^	Ion channel	aCM	
rs7219869	chr17:68337185	KCNJ2*	Roselli et al.^67^	Ion channel		vCM
rs4951258	chr1:205691316	SLC41A1	Nielsen et al.^68^	Ion channel		
rs10741807	chr11:20011445	NAV2	Roselli et al.^67^	Ion channel		
rs10006327	chr4:103890980	SLC9B1	Roselli et al.^67^	Ion channel		
rs6790396	chr3:38771925	SCN10A*	Roselli et al.^67^	Ion channel	aCM	
rs337705	chr5:113737062	KCNN2	Christophersen et al.^65^	Ion channel		aCM
rs76097649	chr11:128764570	KCNJ5	Christophersen et al.^65^	Ion channel	aCM	
rs1545300	chr1:112464004	KCND3	Nielsen et al.^68^	Ion channel		
rs7789146	chr7:150661409	KCNH2*	Roselli et al.^67^	Ion channel		
rs74022964	chr15:73677264	HCN4*	Ellinor et al.^63^	Ion channel	aCM	aCM
rs72700114	chr1:170193825	LINC01142	Nielsen et al.^68^	Noncoding		
rs3176326	chr6:36647289	PANDAR	Nielsen et al.^68^	Noncoding		
rs67969609	chr2:145760353	TEX41	Nielsen et al.^68^	Noncoding		
rs12648245	chr4:174641184	HAND2-AS1	Roselli et al.^67^	Noncoding		
rs7269123	chr20:61157939	C20orf166	Roselli et al.^67^	Noncoding		
rs2145274	chr20:6572014	CASC20	Roselli et al.^67^	Noncoding		
rs2834618	chr21:36119111	LINC01426	Nielsen et al.^68^	Noncoding		aCM
rs9506925	chr13:23368943	LINC00540	Roselli et al.^67^	Noncoding		
rs9506925	chr13:23368943	LINC00621	Nielsen et al.^68^	Noncoding		aCM
rs4963776	chr12:24779491	LINC00477	Nielsen et al.^68^	Noncoding		
rs2738413	chr14:64679960	MIR548AZ	Nielsen et al.^68^	Noncoding		
rs2288327	chr2:179411665	MIR548N	Nielsen et al.^68^	Noncoding		
rs146518726	chr1:51535039	MIR6500	Nielsen et al.^68^	Noncoding		
rs6841049	chr4:83910712	LIN54	Miyazawa et al.^69^	Nuclear		
rs7834729	chr8:21821778	XP07	Roselli et al.^67^	Nuclear		
rs6742276	chr2:61768745	XP01	Roselli et al.^67^	Nuclear		
rs202030113	chr6:152466619	SYNE1	Miyazawa et al.^69^	Nuclear		
rs6747542	chr2:70106832	GMCL1	Nielsen et al.^68^	Nuclear		
rs8096658	chr18:77156537	NFATC1	Miyazawa et al.^69^	Nuclear		
rs7766436	chr6:22598259	HDGFL1	Miyazawa et al.^69^	Nuclear		
rs60212594	chr10:75414344	MYOZ1	Nielsen et al.^68^	Sarcomere		aCM
rs7096385	chr10:69664881	MYPN	Nielsen et al.^68^	Sarcomere		
rs60212594	chr10:75414344	SYNPO2L	Ellinor et al.^63^	Sarcomere		
rs2288327	chr2:179411665	TTN	Christophersen et al.^65^	Sarcomere		
rs2040862	chr5:137419989	FAM13B	Nielsen et al.^68^	Sarcomere		
rs2040862	chr5:137419989	MYOT	Nielsen et al.^68^	Sarcomere	vCM	
rs9284324	chr16:15902715	MYH11	Miyazawa et al.^69^	Sarcomere	vCM	aCM
rs133902	chr22:26164079	MYO18B	Nielsen et al.^68^	Sarcomere		
rs422068	chr14:23864804	MYH6*	Nielsen et al.^68^	Sarcomere		aCM
rs422068	chr14:23864804	MYH7	Roselli et al.^67^	Sarcomere	vCM	vCM
rs73205368	chrX:23399501	PTCHD1	Miyazawa et al.^69^	Signaling		
rs11156751	chr14:32990437	AKAP6	Roselli et al.^67^	Signaling		
rs4896104	chr6:135119089	ALDH8A1	Miyazawa et al.^69^	Signaling		
rs7225165	chr17:1309850	CRK	Nielsen et al.^68^	Signaling		
rs7650482	chr3:12841804	CAND2	Sinner et al.^64^	Signaling		
rs55734480	chr7:14372009	DGKB	Roselli et al.^67^	Signaling	vCM	aCM
rs6771054	chr3:89489529	EPHA3	Nielsen et al.^68^	Signaling	aCM	
Mapgen		FGF9	Selewa et al.^78^	Signaling	vCM	
rs1458038	chr4:81164723	FGF5	Nielsen et al.^68^	Signaling		vCM
rs7612445	chr3:179172979	GNB4	Roselli et al.^67^	Signaling		
rs2040862	chr5:137419989	NPY6R	Nielsen et al.^68^	Signaling		
rs55985730	chr7:128417044	OPN1SW	Nielsen et al.^68^	Signaling		
rs10845620	chr12:12886027	GPR19	Miyazawa et al.^69^	Signaling		
rs12512502	chr4:71776935	MOB1B	Miyazawa et al.^69^	Signaling		
rs1044258	chr10:103605714	C10orf76	Roselli et al.^67^	Signaling		
rs4970418	chr1:918617	PLEKHN1	Miyazawa et al.^69^	Signaling		
rs9953366	chr18:46474192	SMAD7	Roselli et al.^67^	Signaling	aCM	aCM
rs4935786	chr11:121661507	SORL1	Nielsen et al.^68^	Signaling		
rs10753933	chr1:203026214	PPFIA4	Lee et al.^66^	Signaling		
rs1563304	chr17:44874453	WNT3	Nielsen et al.^68^	Signaling		
rs2040862	chr5:137419989	WNT8A	Ellinor et al.^63^	Signaling		
Mapgen		LRIG1	Selewa et al.^78^	Signaling		
rs35544454	chr2:213266003	ERBB4	Nielsen et al.^68^	Signaling	aCM	
rs9899183	chr17:7452977	TNFSF12	Roselli et al.^67^	Signaling		
rs71454237	chr12:70013415	LRRC10	Nielsen et al.^68^	Structural	vCM	
rs7225165	chr17:1309850	MYO1C	Nielsen et al.^68^	Structural		
rs12604076	chr17:76773638	CYTH1	Nielsen et al.^68^	Structural		
rs73241997	chr14:35173775	CFL2	Roselli et al.^67^	Structural		
rs56181519	chr2:175555714	WIPF1	Roselli et al.^67^	Structural		
rs60212594	chr10:75414344	AGAP5	Nielsen et al.^68^	Structural		
rs2727757	chr7:105612736	CDHR3	Miyazawa et al.^69^	Structural		vCM
rs11773845	chr7:116191301	CAV1*	Ellinor et al.^63^	Structural	aCM	aCM
rs11773845	chr7:116191301	CAV2	Nielsen et al.^68^	Structural	aCM	
rs17380837	chr12:26345526	SSPN	Roselli et al.^67^	Structural		
rs7529220	chr1:22282619	HSPG2	Nielsen et al.^68^	Structural		
rs12245149	chr10:65321147	REEP3	Roselli et al.^67^	Structural		
rs79187193	chr1:147255831	GJA5*	Roselli et al.^67^	Structural	aCM	aCM
rs147301839	chr15:57924714	MYZAP	Nielsen et al.^68^	Structural		
rs12809354	chr12:32978437	PKP2	Roselli et al.^67^	Structural		
rs3822259	chr4:10118745	WDR1	Roselli et al.^67^	Structural		
rs12298484	chr12:124418674	DNAH10	Roselli et al.^67^	Structural		
rs35006907	chr8:125859817	MTSS1	Roselli et al.^67^	Structural	aCM	
rs2738413	chr14:64679960	SYNE2	Ellinor et al.^63^	Structural	aCM	
rs10804493	chr3:111554426	PHLDB2	Roselli et al.^67^	Structural	vCM	vCM
rs17005647	chr3:69406181	FRMD4B	Nielsen et al.^68^	Structural	aCM	aCM
rs10213171	chr4:148937537	ARHGAP10	Roselli et al.^67^	Structural		
rs464901	chr22:18597502	TUBA8	Roselli et al.^67^	Structural	vCM	
rs9506925	chr13:23368943	SGCG	Nielsen et al.^68^	Structural		
rs13216675	6q22	GJA1	Sinner et al.^64^	Structural		
rs242557	chr17:44019712	MAPT	Roselli et al.^67^	Structural		
rs4951258	chr1:205691316	NUCKS1	Roselli et al.^67^	Transcription		
rs74884082	chr14:73249419	DPF3	Nielsen et al.^68^	Transcription		
rs2885697	chr1:41544279	SCMH1	Nielsen et al.^68^	Transcription		
rs7096385	chr10:69664881	SIRT1	Nielsen et al.^68^	Transcription		
rs34969716	chr6:18210109	KDM1B	Roselli et al.^67^	Transcription		
rs72926475	chr2:86594487	KDM3A	Roselli et al.^67^	Transcription		
rs2930856	chr12:104471663	HCFC2	Miyazawa et al.^69^	Transcription		
rs9782984	chr1:16199051	SPEN	Miyazawa et al.^69^	Transcription		
rs12591736	chr15:70454139	TLE3	Roselli et al.^67^	Transcription	aCM	aCM
rs6560886	chr12:133150210	FBRSL1	Nielsen et al.^68^	Transcription		
rs284277	chr1:10790797	CASZ1	Roselli et al.^67^	Transcription		
rs35620480	chr8:11499908	GATA4*	Nielsen et al.^68^	Transcription		
rs35005436	chr7:74134911	GTF2I	Roselli et al.^67^	Transcription		
rs12648245	chr4:174641184	HAND2	Lee et al.^66^	Transcription	vCM	
rs1886512	chr13:74520186	KLF12	Miyazawa et al.^69^	Transcription		
rs2274115	chr9:139094773	LHX3	Nielsen et al.^68^	Transcription		
rs6891790	chr5:172670745	NKX2-5*	Roselli et al.^67^	Transcription		
rs9899183	chr17:7452977	SOX15	Nielsen et al.^68^	Transcription		aCM
rs4963776	chr12:24779491	SOX5	Nielsen et al.^68^	Transcription	aCM	aCM
rs12810346	chr12:115091017	TBX3	Roselli et al.^67^	Transcription		
rs883079	chr12:114793240	TBX5*	Sinner et al.^64^	Transcription	aCM	aCM
rs2359171	chr16:73053022	ZFHX3*	Benjamin et al.^62^	Transcription		
rs1891095	chrx:137418967	ZIC3	Miyazawa et al.^69^	Transcription		
rs4743034	chr9:109632353	ZNF462	Roselli et al.^67^	Transcription		
rs1055894680	chr16:30619745	ZNF689	Miyazawa et al.^69^	Transcription		
Mapgen		DBX1	Selewa et al.^78^	Transcription		
Mapgen		ETV1	Selewa et al.^78^	Transcription	aCM	
rs6462079	chr7:28415827	CREB5	Roselli et al.^67^	Transcription	vCM	
rs2738413	chr14:64679960	ESR2	Nielsen et al.^68^	Transcription	aCM	
rs35005436	chr7:74134911	GTF2IRD2	Nielsen et al.^68^	Transcription		
rs13195459	chr6:122403559	HSF2	Nielsen et al.^68^	Transcription		
rs67249485	chr4:111699685	PITX2*	Gudbjartsson et al.^60^	Transcription	aCM	aCM
rs6580277	chr5:142818123	NR3C1	Roselli et al.^67^	Transcription		
Mapgen		BEND5	Selewa et al.^78^	Transcription	aCM	
rs1769758	chr10:80898969	ZMIZ1	Miyazawa et al.^69^	Transcription		
rs72811294	chr17:12618680	MYOCD	Roselli et al.^67^	Transcription		
rs12908004	chr15:80676925	ARNT2	Roselli et al.^67^	Transcription	aCM	
rs73041705	chr3:24463235	THRB	Roselli et al.^67^	Transcription		vCM
rs2286466	chr16:2014283	RPS2	Roselli et al.^67^	Transcription		
rs140185678	chr16:2003016	RPL3L	Nielsen et al.^68^	Transcription	vCM	vCM
rs11881441	chr19:48142746	NOP53	Miyazawa et al.^69^	Transcription		
Mapgene		ZEB2	Selewa et al.^78^	Transcription		
rs3903239	1q24	PRRX1	Ellinor et al.^63^	Transcription		
Mapgen		TAB2	Selewa et al.^78^	Ubiquitination		
rs10873298	chr14:77426525	IRF2BPL	Roselli et al.^67^	Ubiquitination		
rs35569628	chr13:113872712	CUL4A	Nielsen et al.^68^	Ubiquitination		
rs11598047	chr10:105342672	NEURL1	Sinner et al.^64^	Ubiquitination		
rs1344543	chr12:110082115	UBE3B	Miyazawa et al.^69^	Ubiquitination		
rs18758553 0	chr1:10167425	UBE4B	Roselli et al.^67^	Ubiquitination		
rs17303101	chr9:119181794	TRIM32	Miyazawa et al.^69^	Ubiquitination		
rs62521286	chr8:124551975	FBX032	Roselli et al.^67^	Ubiquitination	aCM	
rs7170477	chr15:64103777	HERC1	Nielsen et al.^68^	Ubiquitination		
rs11125871	chr2:61470126	USP34	Roselli et al.^67^	Ubiquitination		
rs62011291	chr15:63800013	USP3	Roselli et al.^67^	Ubiquitination		
rs12604076	chr17:76773638	USP36	Nielsen et al.^68^	Ubiquitination		
rs7508	chr8:17913970	ASAH1	Christophersen et al.^65^	Protein binding		
rs10821415	chr9:97713459	C9orf3	Ellinor et al.^63^	Aminopeptidase O		
rs12426679	chr12:76237987	PHLDA1	Roselli et al.^67^	Phosphatidylinositol phosphate binding, protein binding		
rs517938	chr11:95089882	SESN3	Miyazawa et al.^69^	Leucine binding, protein binding		aCM
rs12245149	chr10:65321147	NRBF2	Nielsen et al.^68^	Protein binding		
rs778479352	chrx:137790580	FGF13	Miyazawa et al.^69^	Sodium channel	vCM	vCM
rs56201652	chr7:92278116	CDK6	Nielsen et al.^68^	Cell cycle		
rs3176326	chr6:36647289	CDKN1A	Roselli et al.^67^	Cell cycle		
rs76460895	chr10:50485434	TMEM273	Miyazawa et al.^69^	Cell membrane		
rs5754508	chr22:21999229	CCDC116	Miyazawa et al.^69^	Centrosomal (coiled-coil domain containing 116)		
rs2540949	chr2:65284231	CEP68	Christophersen et al.^65^	Centrosomal		
Mapgen		CHRNB2	Selewa et al.^78^	Acetylcholine-gated monoatomic cation-selective channel activity		
rs10773657	chr12:123327900	HIP1R	Nielsen et al.^68^	Clathrin trafficking network	vCM	aCM
rs62483627	chr7:106856002	COG5	Roselli et al.^67^	Protein binding		
Mapgen		ELOVL6	Selewa et al.^78^	Fatty acid elongase activity		
Mapgen		EFNA5	Selewa et al.^78^	Ephrin receptor binding		
rs2012809	chr5:128190363	SLC27A6	Nielsen et al.^68^	Fatty acid transmembrane transporter activity	vCM	
rs11658278	chr17:38031164	GSDMB	Nielsen et al.^68^	Phosphatidylinositol-4,5-bisphosphate binding		
rs28387148	chr2:127433465	GYPC	Nielsen et al.^68^	Protein binding		
rs2031522	chr6:87821501	CGA	Nielsen et al.^68^	Hormone activity		
rs17430357	chr8:118863412	EXT1	Miyazawa et al.^69^	Metal ion binding		
rs10458660	chr10:77936576	C10orf11	Roselli et al.^67^	Melanocyte differentiation		aCM
rs139557	chr22:42189407	MEI1	Miyazawa et al.^69^	Genomic integrity		
rs2738413	chr14:64679960	MTHFD1	Nielsen et al.^68^	Methylenetetrahydrofolate dehydrogenase (NAD+) activity	vCM	
rs1933723	chr1:100149308	PALMD	Miyazawa et al.^69^	Protein binding	aCM	
rs75414548	chr1:39385714	NDUFS5	Miyazawa et al.^69^	NADH dehydrogenase (ubiquinone) activity		
rs60212594	chr10:75414344	NUDT13	Nielsen et al.^68^	Metal ion binding		
rs4965430	chr15:99268850	IGF1R	Roselli et al.^67^	Insulin-like growth factor receptor activity		
rs34080181	chr3:66454191	SLC25A26	Nielsen et al.^68^	*S*-Adenosyl-l-methionine transmembrane transporter activity		
rs7578393	chr2:26165528	KIF3C	Roselli et al.^67^	Cytoskeletal motor activity		
rs2860482	chr12:57105938	NACA	Roselli et al.^67^	Unfolded protein binding		
rs3176326	chr6:36647289	PI16	Nielsen et al.^68^	Peptidase inhibitor activity		
rs10804493	chr3:111554426	PLCXD2	Nielsen et al.^68^	Phosphoric diester hydrolase activity		vCM
rs73366713	chr6:16415751	ATXN1	Roselli et al.^67^	DNA binding		
rs14651872 6	chr1:51535039	C1orf185	Roselli et al.^67^	Unknown		
rs17118812	chr5:139703286	PFDN1	Miyazawa et al.^69^	Protein folding chaperone		
rs3746471	chr20:36841914	KIAA1755	Miyazawa et al.^69^	Guanyl-nucleotide exchange factor activity		
rs11590635	chr1:49309764	AGBL4	Nielsen et al.^68^	Zinc ion binding		aCM
rs6994744	chr8:141740868	PTK2	Roselli et al.^67^	Protein tyrosine kinase activity		
rs10760361	chr9:127178266	PSMB7	Roselli et al.^67^	Threonine-type endopeptidase activity		
rs34969716	chr6:18210109	DEK	Nielsen et al.^68^	DNA binding		
rs147301839	chr15:57924714	GCOM1	Nielsen et al.^68^	Fusion protein		
rs72926475	chr2:86594487	REEP1	Roselli et al.^67^	Microtubule binding		
rs8088085	chr18:48708548	MEX3C	Nielsen et al.^68^	Metal ion binding		
rs9899183	chr17:7452977	FXR2	Nielsen et al.^68^	RNA binding		
rs3951016	chr6:118559658	SLC35F1	Nielsen et al.^68^	Transmembrane transporter activity	vCM	vCM
rs3820888	chr2:201180023	SPATS2L	Roselli et al.^67^	RNA binding	aCM	
rs11658278	chr17:38031164	ORMDL3	Nielsen et al.^68^	Protein binding		
rs10749053	chr10:112576695	RBM20	Nielsen et al.^68^	RNA binding		
rs10500790	chr11:14036189	SPON1	Miyazawa et al.^69^	Extracellular matrix structural constituent	aCM	
rs34080181	chr3:66454191	LRIG1	Roselli et al.^67^	Protein binding		
rs11527634	chr10:32772734	CCDC7	Miyazawa et al.^69^	Tumorogenesis		
rs117984853	chr6:149399100	UST	Roselli et al.^67^	Sulfotransferase activity		
rs12209223	chr6:76164589	FILIP1	Miyazawa et al.^69^	Cytoskeletal protein		
rs60902112	chr3:194800853	XXYLT1	Nielsen et al.^68^	Xylosyl transfer activity		
rs11658278	chr17:38031164	ZPBP2	Nielsen et al.^68^	Transcription		
rs35005436	chr7:74134911	LOC101926943	Nielsen et al.^68^	Noncoding		
rs6596717	chr5:106427609	LOC102467213	Nielsen et al.^68^	Noncoding		
rs72700118	1q24	Mettl11b	Christophersen et al.^65^	N-terminal methylation		

Genes were linked to SNPs by the cited publication. Mapgen (Selewa et al.^78^) predicts AF-associated genes through compiling epigenetic features of cardiomyocytes with SNP information. Gene categories were determined through literature searches.

A subset of genes (*) are also implicated by inherited rare variants in AF pedigrees or targeted sequencing studies.

We annotated the main functional classes of AF-associated genes and their chamber-selective expression (Fig. 2b). Ion channel genes were significantly enriched for aCM-selective expression, while Ca^2+^-handling genes frequently exhibited vCM-selective expression. These results highlight the importance of ion channels to regulate the atrial action potential and maintain rhythm. While AF genes were more often aCM-selective than vCM-selective, most AF genes were not aCM-selective (Fig. 2a; Table 1).

Since TBX5 is a master regulator of aCM identity^21,24^, we also examined if genes implicated in AF are downstream of TBX5 based on our recently published single-cell multiome dataset of TBX5 knockout and control atria^24^. Almost a third of GWAS-implicated genes (64, 29.6%) were significantly differentially expressed in TBX5 knockout aCMs. This striking number included seven of the ten genes that showed aCM-selectivity in both mice and humans. Amongst scored genes, the PIP score for TBX5 targets was significantly higher than non-targets (0.68 vs. 0.56). However, the proportion of genes that did and did not receive a score did not significantly differ between TBX5 targets and non-targets. A greater number of genes regulated by TBX5 were downregulated upon TBX5 inactivation (39 of 64, 61%) than upregulated (25 of 64, 39%), consistent with TBX5’s general function as a transcriptional activator. Overall, these data highlight the important role of TBX5 in regulating atrial gene expression and maintaining normal atrial rhythm.

### Rare, large-effect AF variants

While GWAS studies focus on common genetic variants, often with small effect sizes, rare variants causing AF have been described, often in AF pedigrees. The genetics of familial AF were recently reviewed^80^. Overall, familial AF studies have implicated 38 genes; 13 of these genes were also implicated by GWAS, whereas 25 were uniquely identified through pedigree studies (Tables 1 and 2)^80^. In contrast to SNPs identified by GWAS, which are mostly noncoding and affect a variety of different gene categories (Fig. 2b), familial AF variants typically occur in protein-coding regions and predominantly affected genes encoding ion channels (Table 2). Many affected genes were selectively expressed in aCMs (nine genes) or vCMs (eight genes) in either mice or humans (Table 2), and chamber-selective genes were not enriched in specific functional categories (Fig. 2c). A smaller percentage of these genes (6, 16%) were downstream targets of TBX5^24^ compared to the nearly 30% of genes identified by GWAS.

**Table 2:** Genes implicated in AF by targeted sequencing approaches and studies of AF pedigrees

Position (hg19)	Prioritized gene	Category	Original citation	Chamber selective (M.m)	Chamber selective (H.s.)
1q43	RYR2	Ca^2+^	Zhabyeyev et al.^123^		
chr21	KCNE1	Ion channel	Olesen et al.^109^	vCM	
chr21	KCNE2	Ion channel	Yang et al.^102^	vCM	
chr12	CACNA2D4	Ion channel	Zhang et al.^157^		
chr10	CACNB2	Ion channel	Zhang et al.^157^		
chr12	KCNJ8	Ion channel	Delaney et al.^105^		vCM
chr12	KCNA5	Ion channel	Christophersen et al.^158^	vCM	aCM
chr11	KCNQ1	Ion channel	Hong et al.^159^		
chr11	KCNE3	Ion channel	Lundby et al.^103^		
chr11	KCNE5	Ion channel	Ravn et al.^104^		
chr19	SCN1B	Ion channel	Olesen et al.^109^	vCM	aCM
chr11	SCN2B	Ion channel	Watanabe et al.^110^		
chr11	SCN3B	Ion channel	Olesen et al.^111^	aCM	
chr11	SCN4B	Ion channel	Li et al.^112^	vCM	vCM
chr12	ABCC9	Ion channel	Olson et al.^160^	vCM	vCM
chr1	LMNA	Nuclear	Pan et al.^161^		
chr5	NUP155	Nuclear	Zhang et al.^162^		
chr20	JPH2	Sarcomere	Beavers et al.^124^		
chr1	NPPA	Signaling	Ren et al.^163^	aCM	aCM
chr1	GREM2	Signaling	Müller et al.^164^		
chr6	GJA1	Structural	Thibodeau et al.^120^		
chr20	GATA5	Transcription factor	Wang et al.^165^	aCM	aCM
chr18	GATA6	Transcription factor	Li et al.^133^		
chr8	NKX2-6	Transcription factor	Wang et al.^89^		
chr3	SHOX2	Transcription factor	Li et al.^87^	aCM	aCM

## AF pathophysiological mechanisms driven by altered gene expression programs

Mechanisms of AF pathophysiology have been intensely investigated, and yet remain incompletely understood^81–83^. In properly functioning atria, aCMs contract coordinately and only in response to electrical impulses from the sinoatrial node. In AF, re-entrant circuits within the atria permit sustained and chaotic electrical activity. This requires a substrate vulnerable to re-entry plus frequent initiating signals. Factors that create a vulnerable substrate include heterogeneity of conduction velocity or repolarization^84^; unidirectional conduction block; and reduction of minimum path length for re-entry^85^, which is the product of effective refractory period and conduction velocity. Initiating signals include ectopic foci and excessive Ca^2+^ release leading to triggered activity^85^. In this section, we link altered gene expression and AF genetic lesions to these fundamental fibrillogenic mechanisms.

### Gene regulation

As previously discussed, a genetic program maintains aCM identity. While TBX5 has been the only TF reported to be critical for aCM identity maintenance^24^ thus far, many other TFs are expressed in aCMs^86^, and TFs were the most common gene type associated with AF GWAS loci (Table 1). Mutations in several TF genes result in autosomal dominant AF inheritance, including *GATA4*, *GATA5*, *GATA6*, *ZFHX3*, *NKX2-5*, *NKX2-6*, and *PITX2c*^80^. *TBX5* and *SHOX2* mutations also drive familial AF^52,53,87^.

Study of mouse models with perturbed expression of TF genes in aCMs enables identification of key aCM genes that participate in AF pathogenesis, since one can identify the genes directly regulated by mutated TFs. For example, *Tbx5* inactivation in mice causes spontaneous AF and altered the expression of genes encoding ion channels and Ca^2+^ handling, creating both the trigger and substrate for AF^23,28^. *Tbx5* knockout aCMs had markedly prolonged action potentials and increased afterdepolarizations, which favor ectopic and triggered activity. These changes in action potential characteristics were linked to reduced expression of TBX5 target genes *SERCA2A* and *RYR2*, which govern Ca^2+^ handling, and multiple potassium channels, which promote repolarization. *Tbx5* knockout aCMs also expressed less *SCN5A* and *CX43*, which would facilitate re-entry by reducing conduction velocity. *Pitx2c* inactivation made mice susceptible to pacing-induced AF by altering the expression of similar target genes^88^. However, since PITX2c is a transcriptional repressor, its inactivation increased expression of many of the same genes, including *SERCA2A*, *RYR2*, and *CX43*^28,88^. Indeed, heterozygosity for either *Tbx5* or *Pitx2c* resulted in AF inducibility that was reduced in *Tbx5* and *Pitx2c* double heterozygous mice^28^. These results indicate that distinct and even opposite perturbations of atrial gene expression can both cause AF.

While mouse models lacking *Pitx2c* consistently demonstrated ECG changes resembling AF or higher AF vulnerability and were recently reviewed^81^, the mechanisms by which Pitx2c inactivation predisposes to AF remain uncertain due to inconsistencies between studies and between models. In humans, a damaging *PITX2c* variant was linked to lone AF in a pedigree^89^. GWAS strongly linked a region near *PITX2* to AF, yet whether this region acts by altering PITX2 protein levels in aCMs remains to be demonstrated^90^. Recent work profiling human atrial development using single nucleus multiomics and spatial transcriptomics demonstrates that *PITX2* is highly expressed in aCMs during embryonic development, and downregulated in mature aCMs, suggesting the strong GWAS signal associated with *PITX2* might be associated with developmental abnormalities that enhance AF risk^91^.

Cellular protein levels do not always closely correlate with levels of the mRNA transcript that encodes them^92^, and post-translational regulatory mechanisms can further alter protein activity. Therefore, another critical question is how AF alters the aCM proteome. A recent study used mass spectrometry to compare a mouse model of physiological hypertrophy (IGF1R overexpression), which did not exhibit atrial dysfunction, to a model of pathological hypertrophy (overexpression of Mst1 and dominant negative PI3K), which developed dilated cardiomyopathy with fibrotic remodeling of the atria and impaired atrial contractility^93^. Differential analysis between these models identified the downregulation of proteins associated with mitochondria, metabolism, and heart contraction in atria of the pathological hypertrophy model. Comparative analysis of the pathological model to proteins differentially identified in human AF showed conservation of changes in fatty acid metabolism, heart contraction, and mitochondrial organization.

A mass spectrometry analysis of atrial protein downregulated in a canine tachypacing model of AF similarly showed downregulation of proteins involved in contractility, with corresponding reduced force of contraction, decreased resting tension, and increased calcium sensitivity of AF aCMs^94^. AF samples demonstrated increased degradation of myofilament proteins Titin, MYBPC3, and ACTN2 in a pattern consistent with calpain cleavage. In settings of HF, activation of the Ca^2+^-sensitive calpain protease system results in cleavage of sarcomeric proteins, some of which are known to play cardioprotective roles in the ventricle^95^. Although protein levels of calpain and its endogenous inhibitor calpastatin were unaltered in the AF model, calpain activity was increased, possibly due to elevated intracellular Ca^2+^ in AF^94^.

Together, these studies show that the aCM cell state is profoundly altered in AF at both the transcriptomic and proteomic levels. More work is needed to determine if these alterations are reversible, and how each of the many changes contributes toward the progressive nature of AF.

### Electrical function

aCMs express ion channels that shape their action potential and mediate excitation–contraction coupling. GWAS and coding variants have implicated several potassium channel genes in AF pathogenesis. Potassium channels mediate cardiac repolarization. With a few exceptions, AF-associated potassium channel variants increase potassium currents and therefore shorten action potential duration and effective refractory period, which create a vulnerable substrate by allowing the shorter re-entrant circuits responsible for AF. For example, *KCNJ5* interacts with *KCNJ3* to form the acetylcholine-activated potassium channel (*I*_KACh_), which is specifically expressed in aCMs and was shown to be constitutively active in patients with chronic AF^96^. A KCNJ3-N83H missense variant that increased *I*_KACh_ caused familial bradycardia and AF^97^. Zebrafish expressing KCNJ3-N83H exhibited bradycardia that was rescued by the addition of a selective K^+^ channel inhibitor, NIP-151. In mice, pharmacological stimulation of *I*_KACh_ enhanced AF inducibility, whereas ablation of *KCNJ5* prevented AF^98^. A strong GWAS signal was also observed near *KCNJ5*, suggesting that common variants controlling its expression influence AF susceptibility^67–69^. A pedigree with a missense variant in *KCNQ1*, which encodes the pore-forming subunit of the potassium channel *I*_Ks_^99^, likewise implicated this channel in AF. In this four-generation pedigree, a KCNQ1-S140G missense variant that increased channel activity caused autosomal dominant AF^100^. In addition to these two examples, coding variants have been described as AF drivers for the potassium channels genes *KCNE1*, *KCNE2*, *KCNE3*, *KCNE5*, *KCNJ8*, *KCNA5*, and *KCND3*^101–107^, but the causative role of these variants was less well substantiated due to limitations such as small family sizes or low numbers of affected patients.

Altered expression or activity of other types of channels are also implicated in AF. Variants in both the pore-forming alpha subunit and regulatory beta subunits of the major cardiac sodium channel, responsible for the depolarizing current *I*_Na_, have also been associated with AF^108–112^. *SCN5A* and *SCN4B* have been most directly implicated. One study^108^ directly sequenced the coding region and splice junctions of the entire *SCN5A* gene in 375 patients with AF (118 patients with lone AF), resulting in the identification of 8 novel variants in 10 probands^113^. The identified AF-associated variants were in highly conserved residues and altered SCN5A activity. Six probands reported AF in family members, and pedigree analysis indicated that these variants co-segregated with AF with a dominant inheritance pattern in all six families. A family that exhibited sick sinus syndrome, atrial flutter, and AF was found to have a truncating *SCN5A* variant, R1860Gfs*12, which resulted in a 70% decrease in channel current density when expressed in HEK293 cells^114^. Loss of *I*_Na_ is predicted to slow atrial conduction velocity, reducing the path length required for re-entry and creating a favorable environment for multiple-circuit re-entry^115^. These data are consistent with AF GWAS studies, which implicated a locus containing *SCN5A* and the neighboring gene *SCN10A*. The implicated region functioned as an enhancer element that was activated by TBX5, and the SNP reduced the enhancer’s activity^116^.

Two out of the three small-conductance Ca^2+^-activated K^+^ (SK) channels, encoded by *KCNN2* and *KCNN3*, were implicated in AF by human genetic studies^61,65^ and have been shown to have enhanced channel function in humans with chronic AF^117^. Although protein levels of the channels were not different in RA cardiomyocytes between control and chronic AF patients, SK2 membrane localization was enhanced in AF patients and channel activity was increased due to decreased calmodulin phosphorylation mediated by PP2A. Recently, an SK2 channel inhibitor AP30663 met the primary endpoint of cardioversion occurring within 1 h of administration in a phase 2 clinical trial, without serious treatment-related adverse events^117^. These exciting results point to the potential efficacy of SK channel inhibition as a novel antiarrhythmic approach for AF.

Together, these data show that proper control of electrical signaling in aCMs is functionally required for rhythm maintenance. Altered ion channel functionality affects atrial conduction velocity and the effective refractory period of aCMs.

### Communication between neighboring aCMs

An important ultrastructural feature of aCMs, as well as vCMs, is the intercalated disc (ICD), which is located on the ends of CMs at their junction with neighboring CMs. The ICD contains desmosome and cadherin complexes that physically couple neighboring CMs, and gap junction channels that support the propagation of action potentials between neighboring CMs^118^. There are two major gap junction proteins expressed in the aCMs: *GJA1*, which is expressed in both aCMs and vCMs, and *GJA5*, which is aCM-selective^119^. *GJA5* neighbors a GWAS locus associated with AF risk^67^. Loss-of-function somatic mutations in both *GJA1* and *GJA5* have further implicated these genes in AF^119,120^. For example, genomic DNA sequencing of atrial tissue and peripheral blood lymphocytes of 15 AF patients revealed *GJA5* variants in the heart tissue of 4 patients. The variants were absent in the blood of three of the four patients, consistent with somatic mutation. Interestingly, both GJA1 and GJA5 are downregulated in mice with cardiomyocyte inactivation of *Tbx5* or *Lkb1*, two genetic mouse models with spontaneous AF^24,28,121,122^. These data underscore the importance of these gap junctions for supporting normal atrial rhythm, probably through supporting inter-aCM electrical coupling. Disrupting aCM electrical coupling could predispose to re-entry by reducing conduction velocity and increasing conduction velocity heterogeneity within the atrial myocardium.

### Excitation–contraction coupling

Coordinated atrial contractions promote the flow of blood through the atria and into the ventricles. Fibrillating atria fail to contract uniformly, and individual aCMs have decreased contractile force, resulting in blood pooling and increased risk of thrombosis and stroke. During excitation–contraction coupling, sarcolemmal depolarization opens voltage-gated L-type calcium channels. The influx of Ca^2+^ signals to the ryanodine receptor, RYR2, to release Ca^2+^ stored in the sarcoplasmic reticulum, producing a Ca^2+^ transient that interacts with sarcomeric proteins and stimulates contraction. During diastole, SERCA2A pumps cytoplasmic Ca^2+^ back into the sarcoplasmic reticulum, and NCX1, a sodium–calcium exchanger on the sarcolemma, returns Ca^2+^ to the extracellular space. Many proteins involved in this intricate process are genetically implicated in AF, including RYR2^123^, JPH2^124^, PLN^65^, CASQ2^67^, and CAMK2D^67^ (Tables 1 and 2).

Mutations in RYR2 are most commonly associated with CPVT. In addition to hallmark exercise-induced ventricular tachycardia, some patients have atrial arrhythmias including AF^125^. This is modeled in mice with CPVT-causing RYR2 variants, which have increased AF vulnerability^126,127^. These experimental and clinical observations clearly implicate Ca^2+^ handling as an important mechanism in AF. Leaky RYR2 channels result in increased diastolic Ca^2+^ leak^128^, which activates Ca^2+^-calmodulin dependent kinase 2 (CAMK2), a key mediator of the fight-or-flight response whose chronic activation contributes to pathological cardiac remodeling and arrhythmias^129^. Activated CAMK2 phosphorylates several Ca^2+^ handling proteins including RYR2, creating a positive feedback loop that increases aberrant RYR2 Ca^2+^ release. Elevated diastolic Ca^2+^ promotes electrogenic Na^+^–Ca^2+^ exchange by NCX1, leading to delayed afterdepolarizations and triggered activity^130^. These afterdepolarizations also cause dispersion of repolarization and refractoriness, increasing susceptibility to re-entry^131^. The importance of CAMK2-mediated phosphorylation of RYR2 is highlighted by studies of mice in which the CAMK2 phosphorylation site on RYR2 (serine 2814) has been ablated. These RYR2-S2814A mice were protected from AF triggered by genetic or environmental insults^132,133^. In addition to RYR2, CAMK2 also phosphorylates PLN to relieve PLN-mediated SERCA2 inhibition^134^, resulting in increased SR calcium loading that is further enhanced by the aCM depolarization frequency in AF^135^. HF patients had reduced RYR2 protein in atrial myocardium, and relative Ser2814-RyR2 phosphorylation was greater in the subset with chronic AF^136^. Increased CAMK2-mediated RYR2 phosphorylation was also shown to occur in patients experiencing AF following open heart surgery, a complication mediated by inflammatory signaling (see below) that affects ~30% of adult cardiac surgery patients^137^. Together, these studies indicate that excessive diastolic RYR2 activity creates favorable conditions for AF^138^.

AF was originally described as predominantly a disease affecting the electrical function of the heart. Yet family studies and GWAS both link structural genes to AF, including the alpha and beta myosin heavy chain isoforms MYH6^139^ and MYH7^140^, and TTN, which connects the Z-disk to the M-line in sarcomeres and functions as a molecular spring to imbue the sarcomere with elasticity^67–69^. Mutations in these genes commonly result in inherited cardiomyopathy^141,142^. The clinical connection between AF and cardiomyopathy is well appreciated^143^, and recent experimental evidence has begun to elucidate the molecular mechanisms linking the conditions at the transcriptional level^144^. Intriguingly, mutations in genes commonly associated with HF have been linked with early onset, lone AF. A recent study^145^ examining a cohort of 1293 patients with lone AF diagnosed prior to age 66 found that pathogenic variants in genes associated with inherited cardiomyopathy syndromes were more common than variants in genes associated with inherited arrhythmias and were observed at greater frequencies in younger lone AF patients. Other recent targeted approaches have shown that loss-of-function mutations in TTN are highly prevalent in individuals with early AF diagnoses^146–148^. One study^146^ performed whole exome sequencing in families with at least three individuals affected with AF and observed an enrichment for TTN truncating mutations (*P* = 1.76 × 10^−6^). This finding was replicated in an additional cohort of 399 lone AF patients (odds ratio = 36.8; *P* = 4.13 × 10^−6^). The causal link between titin truncating variants and AF was further supported by the study of heterozygous *ttn* N-terminal truncation in zebrafish, which caused sarcomeric defects, irregularly irregular heart rhythm, and increased atrial fibrosis.

These data support an evolving view of AF as the result of atrial myopathy as well as electrical dysfunction.

### Inflammatory signaling

Recent work has demonstrated inflammatory signaling as an important mechanism promoting AF^149^. At least two different inflammatory mechanisms have been identified, including activation of the NLRP3 (NACHt-, LRR-, and pyrin domain-containing 3) inflammasome within aCMs^150^, and the infiltration of CCR2^+^ proinflammatory macrophages into diseased atrial myocardium, which promotes pathological atrial remodeling^151^.

The NLRP3 inflammasome controls the production of inflammatory cytokines within cells through the cleavage of pro-IL-1β and pro-IL-18, which are released into the extracellular environment through membrane pores formed by the N terminus of gasdermin D (GSDMD-NT), which is also produced by inflammasome cleavage^152^. Recent work^150^ examining protein levels of NLRP3, the inflammasome component ASC, and the active form of its effector protein pro-caspase1, revealed their upregulation in aCMs of chronic AF patients. Furthermore, overexpression of constitutively activated NLRP3 in cardiomyocytes increased AF vulnerability. The enhanced vulnerability was associated with an abbreviated atrial effective refractory period and fibrotic atrial remodeling. This mechanism was also shown to participate in post-operative AF by promoting CAMK2-mediated RYR2 phosphorylation of Ser2814^137^, as mentioned previously.

Inflammatory signaling in the malfunctioning atria results in the recruitment of CCR2^+^ SPP1^+^ macrophages in human AF patients^151^. Expansion of this proinflammatory macrophage population was similarly observed in a mouse model of enhanced AF vulnerability produced by combining hypertension, obesity and mitral valve regurgitation (HOMER mice), risk factors driving AF in humans. SPP1, also known as osteopontin, is a matricellular protein that stabilizes collagen^153^ and was shown to promote fibrosis in hypertensive mice^154^. Transplanting *Spp1*^*−/−*^ bone marrow into wild-type mice that were then subjected to HOMER reduced AF inducibility and fibrotic remodeling. Examining crosstalk between SPP1^+^ macrophages and other cell types using single-cell transcriptomic data revealed signaling between these macrophages and other atrial immune cells and stromal cells expressing SPP1 receptors. In a subsequent study, the authors used an antibody-siRNA conjugate to silence *Spp1* in cardiac macrophages, leading to reduced AF inducibility and atrial fibrosis^155^.

These data reveal that inflammatory signaling both alters the electrical properties of aCMs and promotes remodeling of the atrial substrate to support AF.

## Conclusions

Although developmental processes driving chamber-selective gene expression programs have been appreciated for a number of years, advances in functional genomics have only recently begun to illuminate the molecular mechanisms by which aCMs and vCMs maintain their differences, revealing ESRR and TBX5 as critical network components that facilitate maintenance of vCM and aCM identity, respectively. aCMs and vCMs represent the two major cardiomyocyte subtypes, but even within the same chamber there are multiple cardiomyocyte states with non-identical gene expression profiles^156^. More work will be required to decipher the functional importance of these cardiomyocyte states in cardiac homeostasis and disease.

Large GWAS studies have identified 150 genomic loci that are significantly associated with AF, and continued expansion of these studies to better represent diverse ethnic groups, will likely further increase this number. These GWAS variants are overwhelmingly in non-coding regions that likely function as CREs. Linking these non-coding variants to specific genes is currently an active area of research. About 20% of candidate genes neighboring these GWAS loci are selectively expressed at higher levels in aCMs than vCMs, and the expression of about 30% is regulated by TBX5, a master regulator of aCM identity. We combined this information with scoring systems aimed at identifying causative AF genes to create an encyclopedia of genes with probable roles in AF (Tables 1 and 2). Additional work examining the function of these genes in animal and human PSC-derived models will undoubtedly enhance our understanding of the molecular mechanisms underlying AF.

AF results from genetic lesions that directly affect the electrical and Ca^2+^ handling properties of aCMs. In addition, genetic variants in cardiomyocyte structural components also cause AF. Whether these classes of perturbations cause AF through distinct mechanisms or through a common shared AF pathway is an important unresolved question. If there are distinct mechanisms that lead to AF, how can the primary mechanisms be discerned in individual patients to enable precise, targeted therapy? These types of questions will drive future efforts to understand AF pathophysiology and ultimately improve our ability to control AF in the clinic.

## Supplementary information

Supplementary_Extended_data_1_Deseq2_human

## Data Availability

Mouse aCM and vCM expression data were sourced from Cao et al.^21^ (GSE215065), while human expression data were sourced from Kanemaru et al.^33^ (processed data were downloaded from https://www.heartcellatlas.org/). WT and TBX5 KO mouse aCM snRNAseq data was obtained from Sweat et al.^24^ (GSE222970).
